# Supplementary material for: Impact of Mycobacterium tuberculosis Infection on Human B Cell Compartment and Antibody Responses
Source: Cells. 2022 Sep 17;11(18):2906. doi: 10.3390/cells11182906 (PMC9497247; doi:10.3390/cells11182906)
Supplement: Supplementary file 1 [file cells-11-02906-s001.zip › Supplemental Table S1.pdf]

Supplemental Table S1. List of genes in database (accession number: GSE54992).

	SYMBOL	*RANK IN GENE LIST*	*RANK METRIC SCORE*	*RUNNING ES*	*CORE ENRICHMENT*
B cell differentiation (GO:0030183)	IL10	96	0.758	0.0341	Yes
	IFNA4	179	0.631	0.0624	Yes
	C17orf99	181	0.63	0.0952	Yes
	ADGRG3	319	0.518	0.1145	Yes
	PAX5	328	0.512	0.1408	Yes
	DRD2	466	0.459	0.157	Yes
	IL11	629	0.412	0.1693	Yes
	FZD9	647	0.407	0.1896	Yes
	JAK3	928	0.346	0.1917	Yes
	SP3	960	0.34	0.2077	Yes
	IFNA7	1054	0.324	0.2193	Yes
	BCL6	1485	0.272	0.2091	Yes
	IFNW1	1559	0.264	0.2187	Yes
	IFNA6	1726	0.248	0.2222	Yes
	TNFSF13B	2114	0.217	0.2116	Yes
	NOTCH2	2132	0.216	0.2219	Yes
	NFAM1	2223	0.209	0.2277	Yes
	CDH17	2643	0.182	0.2134	Yes
	FLT3	2690	0.18	0.2202	Yes
	SLAMF8	2743	0.177	0.2265	Yes
	IL4	2942	0.167	0.224	Yes
	IFNA21	3051	0.162	0.2263	Yes
	PIK3R2	3120	0.16	0.2308	Yes
B cell activation (GO:0042113)	BANK1	15023	-0.152	-0.2717	No
	ITFG2	15255	-0.165	-0.2789	Yes
	RAG1	15324	-0.169	-0.2767	Yes
	ITGA4	15400	-0.174	-0.2747	Yes
	MSH2	15472	-0.18	-0.2723	Yes
	NTRK1	15580	-0.187	-0.2716	Yes
	CR2	15705	-0.198	-0.2715	Yes
	CASP8	15709	-0.198	-0.2646	Yes
	LAX1	15777	-0.204	-0.261	Yes
	ZAP70	15791	-0.205	-0.2544	Yes
	NKX2-3	15820	-0.208	-0.2485	Yes
	CD79A	15863	-0.212	-0.2432	Yes
	LEF1	15900	-0.215	-0.2375	Yes
	IL7	16286	-0.258	-0.2501	Yes
	CD180	16341	-0.264	-0.2437	Yes
	RASGRP1	16382	-0.27	-0.2362	Yes
	RAG2	16408	-0.273	-0.2278	Yes

	FCRL1	16444	-0.279	-0.2197	Yes
	CD19	16459	-0.281	-0.2104	Yes
	TPD52	16528	-0.291	-0.2038	Yes
	IFNA2	16548	-0.294	-0.1943	Yes
	MS4A1	16564	-0.296	-0.1845	Yes
	PIK3R1	16605	-0.302	-0.1759	Yes
	CXCR5	16650	-0.31	-0.1672	Yes
	CNOT6	16661	-0.312	-0.1565	Yes
	IRF8	16663	-0.312	-0.1453	Yes
	HDAC9	16987	-0.381	-0.15	Yes
	IL6	17059	-0.401	-0.1396	Yes
	SASH3	17099	-0.414	-0.1269	Yes
	DRD3	17143	-0.427	-0.114	Yes
	CCR6	17361	-0.521	-0.1075	Yes
	CHRNB2	17471	-0.606	-0.0919	Yes
	IL7R	17482	-0.614	-0.0703	Yes
	ATAD5	17592	-0.74	-0.0499	Yes
	RABL3	17612	-0.779	-0.0229	Yes
	IKZF3	17651	-0.848	0.0055	Yes
B cell mediated immunity (GO:0019724)	C1QB	280	0.545	0.0272	Yes
	SERPING1	345	0.506	0.0636	Yes
	CD70	431	0.47	0.0959	Yes
	DRD2	466	0.459	0.1302	Yes
	C3	922	0.347	0.1318	Yes
	C1S	957	0.34	0.1568	Yes
	C1QA	1138	0.312	0.1713	Yes
	FCGR1A	1148	0.311	0.1954	Yes
	C2	1173	0.308	0.2183	Yes
	EXO1	1265	0.297	0.2367	Yes
	BCL6	1485	0.272	0.2457	Yes
	C4A	1632	0.257	0.2578	Yes
B cell homeostasis (GO:0001782)	CAMLG	117	0.731	0.1286	Yes
	DRD2	466	0.459	0.1938	Yes
	BCL2L11	812	0.368	0.2424	Yes
	SPNS2	1233	0.301	0.2743	Yes
	TNFAIP3	1734	0.247	0.2917	Yes
	TNFSF13B	2114	0.217	0.3104	Yes
	SOS1	2504	0.19	0.3235	Yes
