# Supplementary figures and images for: Impact of Mycobacterium tuberculosis Infection on Human B Cell Compartment and Antibody Responses
Source: Cells. 2022 Sep 17;11(18):2906. doi: 10.3390/cells11182906 (PMC9497247; doi:10.3390/cells11182906)

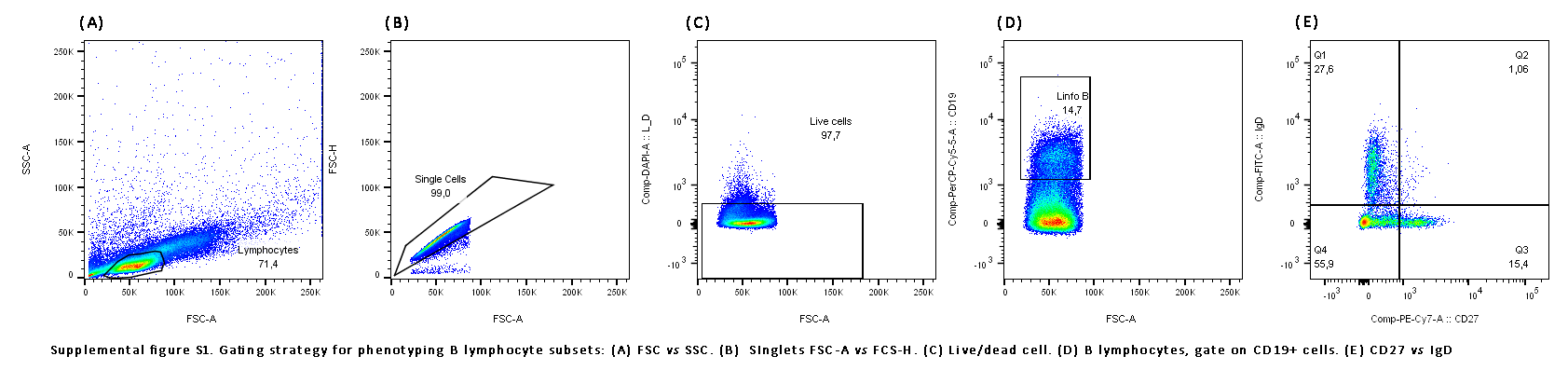

Supplement: Supplementary file 1 [file cells-11-02906-s001.zip › Supplemental Figure S1.tif]

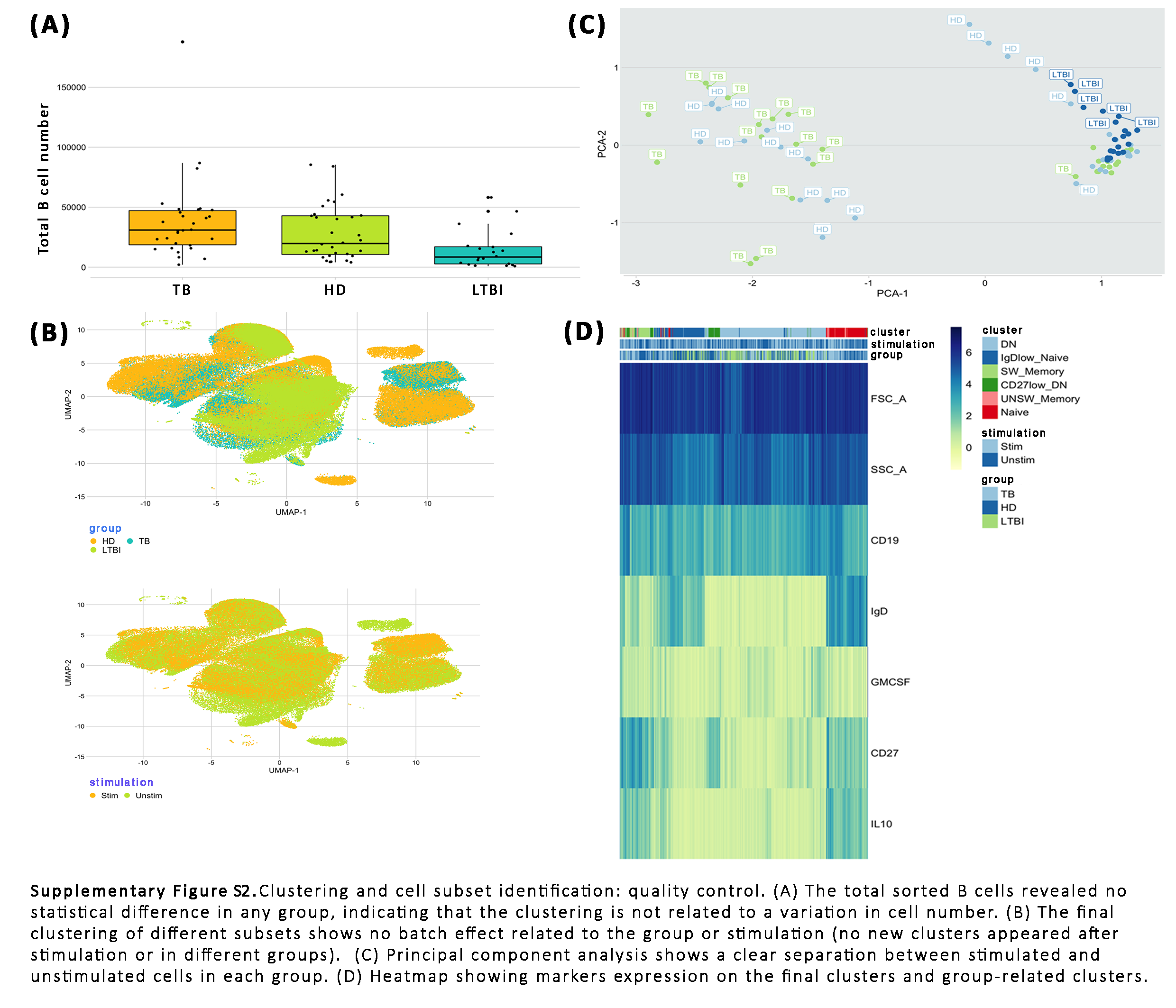

Supplement: Supplementary file 1 [file cells-11-02906-s001.zip › Supplemental Figure S2.tif]
